# Effects of *Zataria multiflora* essential oil on the germinative cells of *Echinococcus granulosus*

**DOI:** 10.1186/s13071-021-04765-8

**Published:** 2021-05-17

**Authors:** Nasim Kowsari, Mohammad Moazeni, Ali Mohammadi

**Affiliations:** 1 Division of Parasitology, Department of Pathobiology, School of Veterinary Medicine, Shiraz University, Shiraz, 71345-1731 Iran; 2 Division of Virology, Department of Pathobiology, School of Veterinary Medicine, Shiraz University, Shiraz, Iran

**Keywords:** *In vitro*, Cell culture, *Echinococcus granulosus*, Germinative cells, *Zataria multiflora*, Essential oil

## Abstract

**Background:**

Novel and more efficient compounds are urgently required for medical treatment of cystic echinococcosis (CE). Germinative cell culture of *Echinococcus granulosus* could be used for anti-echinococcosis agent tests and other biological studies on CE. This study was performed to establish an *in vitro* cell culture model for *E. granulosus* germinative cells and to evaluate the lethal effect of *Zataria multiflora* essential oil (ZMEO) on the cultured cells.

**Methods:**

The inner surface of germinal layers of CE cysts was scraped, and the obtained materials were trypsinized to obtain a suspension of single germinative cells. Medium 199 was used as the basic culture medium and was supplemented with fetal bovine serum, 2-mercaptoethanol, l-cysteine, l-glutamine, glucose, sodium pyruvate, hydatid fluid, amphotericin B and antibiotics. The cells were cultured at a concentration of 10^4^ cells/ml of culture medium and incubated at 37 °C. The culture medium was replaced every 7 days. Chemical composition of ZMEO was identified by GC-MS analysis. ZMEO was tested at concentrations of 0.5–8 mg/ml. Viability of the cells was assessed by trypan blue exclusion assay.

**Results:**

A significant increase in the cell number was evident at 20, 30 and 45 days after cultivation. At 45 days of cultivation, the number of cells was approximately five-fold higher than on the first day. In GC-MC analysis, carvacrol, *p*-cymene, g-terpinene and thymol were found to be the main compounds of ZMEO. The lethal effect of ZMEO on the germinative cells at concentrations of 6, 7 and 8 mg/ml was 100% after 60, 25 and 7 min of exposure, respectively.

**Conclusions:**

At 45 days of cultivation, the cell concentration was suitable for the desired *in vitro* experiments. A high lethal effect of ZMEO on the germinative cells of *E. granulosus* may be considered an opportunity for the introduction of a novel, more effective and safer therapeutic agent for treatment of CE using an herbal product.

**Graphic abstract:**

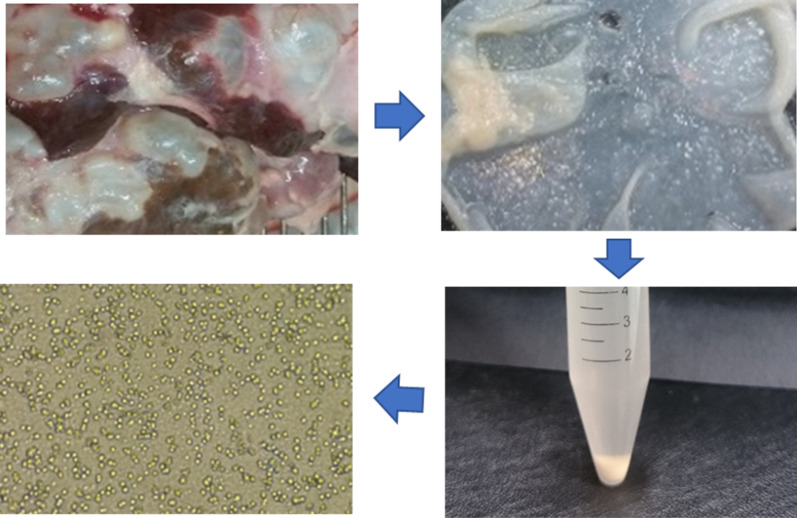

## Background

Cystic echinococcosis (CE), a neglected zoonotic infection with a significant socioeconomic and health consequences, is caused by the larval stage of *Echinococcus granulosus* (*s.l.*) [1]. The parasite life cycle includes dogs and other canids as definitive hosts, while domestic ruminants and humans act as intermediate hosts for the parasite [2]. Infected dogs disseminate the parasite’s eggs into the environment through their feces. Intermediate hosts become infected by ingesting the fertile eggs. Hexacanth embryos are liberated in the small intestine of these hosts, reach the liver and other organs via the blood circulation and develop to the larval form of the parasite (CE cysts). The dogs become infected by eating the infected organs of intermediate hosts [3]. In humans, even though the infection may be clinically silent, it may be severe; in rare cases, it may present as a fatal disease [4].

Number, size, location and viability status of the cysts, the infected organ as well as the bacterial or fungal infection of the cyst may affect the treatment choice. The disease may be treated by surgery, puncture, aspiration, injection, re-aspiration (PAIR), anti-parasitic treatment or watch and wait for inactive cysts [5, 6]. In inoperable cases, anti-parasitic treatment is the only option [6, 7]. In humans, albendazole, as the drug of choice for anti-parasitic treatment of CE [2], should be administered for long periods and at high doses [8]; therefore, it may be accompanied by adverse side effects in the patients [9]. On the other hand, 2 years after the start of treatment, 40% of the cysts may be still in the active status [10]. Hence, generally no complete recovery occurs after treatment with albendazole [5]. Therefore, novel and more efficient compounds are urgently required for medical treatment of CE [11–13].

In addition to the need to evaluate the drug/vaccine efficiency, research on the different aspects of CE is required to study the biological and immunological behavior of the parasite and assess the diagnostic tools for the early detection of the infection in intermediate hosts, particularly in human beings [14, 15].

The larval form of *E. granulosus* (*s.l.*) is a fluid-filled sac consisting of an inner nucleated or germinal layer and an acellular, tough and elastic laminated layer. The cyst is surrounded externally by a host-produced adventitial layer [16]. Reproducing cells of cyst's germinal layer can be applied to establish an *in vitro* cell culture system [17]. As a matter of fact, the germinal layer of the cyst acts as a generous and productive source for the other parts of the cyst including the laminated layer, brood capsules and protoscoleces. Hence, the germinative cell cultures can be used for testing anti-echinococcosis agents [18] as well as for other biological studies on CE. The present study aimed (i) to establish a cell culture model *in vitro* for the germinative cells of the larval stage of *E. granulosus* (*s.l.*) and (ii) to evaluate the lethal effect of *Zataria multiflora* essential oil (ZMEO) on the germinative cells of *E. granulosus in vitro*.

## Methods

### Chemicals

Medium 199 was purchased from Gibco (Brazil). Fetal bovine serum (FBS), sodium pyruvate, penicillin, streptomycin and amphotericin B were obtained from Sigma-Aldrich Co. (Germany). Glucose, l-cysteine, l-glutamine, and 2-mercaptoethanol were purchased from Merck Co. (Germany). ZMEO was obtained from Barij Essence Pharmaceutical Co. (Kashan, Iran). Ten-centimeter cell culture Petri dishes and 12-well plates were purchased from Alian Tajhiz Co. (Shiraz, Iran). All other reagents were of analytical grade.

### Collection of CE cysts

Liver and lungs of livestock infected with CE cysts were aseptically collected from Shiraz (southern Iran) industrial slaughterhouses and transferred to the parasitology laboratory of the School of Veterinary Medicine, Shiraz University, under cold and sterile conditions.

### Isolation of germinative cells

In the laboratory, the liver and lungs containing the cysts were first thoroughly washed with tap water. Then, the cyst surfaces were dried with sterile cotton and gauze and sterilized with 70% ethanol. Subsequently, the fluid of the cysts was aseptically aspirated by sterile syringes. The cyst walls were cut open using a scalpel, and the germinal layer of each cyst was carefully separated from the laminated layer. Germinal layers were washed three times with 40 ml PBS containing 1 ml antibiotics (10,000 μ/ml penicillin and 10 mg/ml streptomycin). Then, the inner surface of the layer was scraped gently by a scraper, and the obtained material was treated with 10 vol. 0.25% trypsin at room temperature for 15 min with continuous shaking (22 cycles/min). Subsequently, the obtained solution was transferred into a sterile glass tube and centrifuged at 715×*g* at 4 °C for 10 min. After discarding the supernatant, the single cell mass remained sedimented at the bottom of the tube. Finally, the cells were counted using a Neubauer chamber before cultivation.

### Preparation of culture medium

The culture medium was prepared based on the study of Albani et al. [19]. Medium 199 was used as the basic culture medium. This medium was supplemented with 10% fetal bovine serum (FBS), cyst fluid (10%), reducing agents including 2-mercaptoethanol (5 × 10^–5^), l-cysteine (100 µM) and l-glutamine (2 mM), glucose (4 mg/ml), sodium pyruvate (1 mM), amphotericin B (0.5 µl/ml) and 100 µg/ml each of penicillin and streptomycin antibiotics. Finally, the pH of the obtained medium was adjusted to 7.5. These supplements were carefully and cautiously added to 250 ml medium 199 inside a laboratory hood and under sterile conditions. The medium was filtered using a syringe filter with 0.22-µm pore size. Finally, the prepared medium was stored in 50-ml Falcon tubes at 4 °C until use.

### *In vitro* culture of germinative cells

The cells were cultured based on the study of Albani et al. [19] with some modifications. Ten-centimeter cell culture Petri dishes and 12-well plates were used as the culture vessels. Ten-centimeter cell culture Petri dishes were filled with 8 ml culture media, and the wells of 12-well plates were filled with 2 ml of the medium. The cells were cultured with a concentration of 10^4^ cells/ml culture medium. Amphotericin B (50 µl/100 ml of culture medium) was added to prevent fungal growth in the culture media. The culture vessels were incubated at 37 °C in an incubator without CO_2_ and in aerobic conditions. The culture medium was replaced every 7 days. Cell cultures were monitored periodically (every 10 days), and photography was performed using an inverted microscope.

### Cell passaging

Two months after the start of cultivation, the attached cells were passaged. In the first step, the culture medium was completely removed, and then the attached cells were washed gently with PBS (2 times). Subsequently, after discarding the used PBS, the attached cells were resuspended in 25% trypsin-containing EDTA (2 mg/ml). Enough solution was added to cover the bottom of the culture plates (1.5 ml). After 15 min incubation at 37 °C, the culture media containing FBS were added to neutralize the trypsin effect. After pipetting, the resuspended cells were transferred to a Falcon tube and centrifuged at 180×*g* for 5 min. Then, the medium was discarded and fresh medium was added. After pipetting, the cell suspension was transferred into the new culture plates. In this study, the cells were passaged four times with 2-month intervals.

### GC and GC-MS analysis of ZMEO

The chemical composition of ZMEO was determined using a gas chromatography (GC model 7890)-mass spectrometry (MS model 5975) system (Agilent Technologies, USA). The analysis was performed using a HP-5 fused-silica column with 30 m length, 0.25 mm diameter and 0.25 μm film thickness. The oven temperature was set to rise from 60 to 210 °C at a rate of 3 °C/min and then was increased to 240 °C at a rate of 20 °C/min. The final temperature was held for 8.5 min. Both the transfer line and injector temperatures were kept at 280 °C. Helium was used as the carrier at a flow rate of 1 ml/mi. The electron ionization energy of MS was 70 eV. The constituents of the oil were identified by comparing their retention indices with those reported in the Wiley GC-MS Library and Adams Library [20].

### *In vitro* lethal effect of ZMEO on germinative cells

ZMEO was used at concentrations of 0.5, 1, 2, 3, 4, 5, 6, 7 and 8 mg/ml. For better solubility of ZMEO in the culture medium, twice as much Tween 80 was added as ZMEO (v/v). The cultured cells were treated with different concentrations of ZMEO at different times. In initial evaluations, lower concentrations of the oil were applied for 12, 24 and 48 h. Accordingly, higher concentrations were applied for shorter times. All the experiments were performed in triplicate, except for 0.5, 1 and 5 mg/ml, which were carried out in duplicate. Albendazole at a concentration of 20 mg/ml for 120 min was used as positive control.

### Viability test

Viability of cells was assessed by trypan blue exclusion assay. Germinative cell suspension (0.1 ml) and 0.25% trypan blue stain (0.1 ml) were placed in a well of a 96-well plate. After gently pipetting, the plate was incubated at room temperature for 5 min. Subsequently, the viable (uncolored) and dead (blue colored) cells were counted using a Neubauer chamber. The number of cells counted in each experiment ranged from 265,000 to 540,000 (average 41,200 cells).

### Statistical analysis

The results of the lethal effect of ZMEO on the cultured cells, are presented as mean ± standard deviation. Statistical analysis was performed using SPSS software, version 22. The data were analyzed by Mann-Whitney *U* test. *P* < 0.05 was considered statistically significant.

## Results

**Fig. 1:**
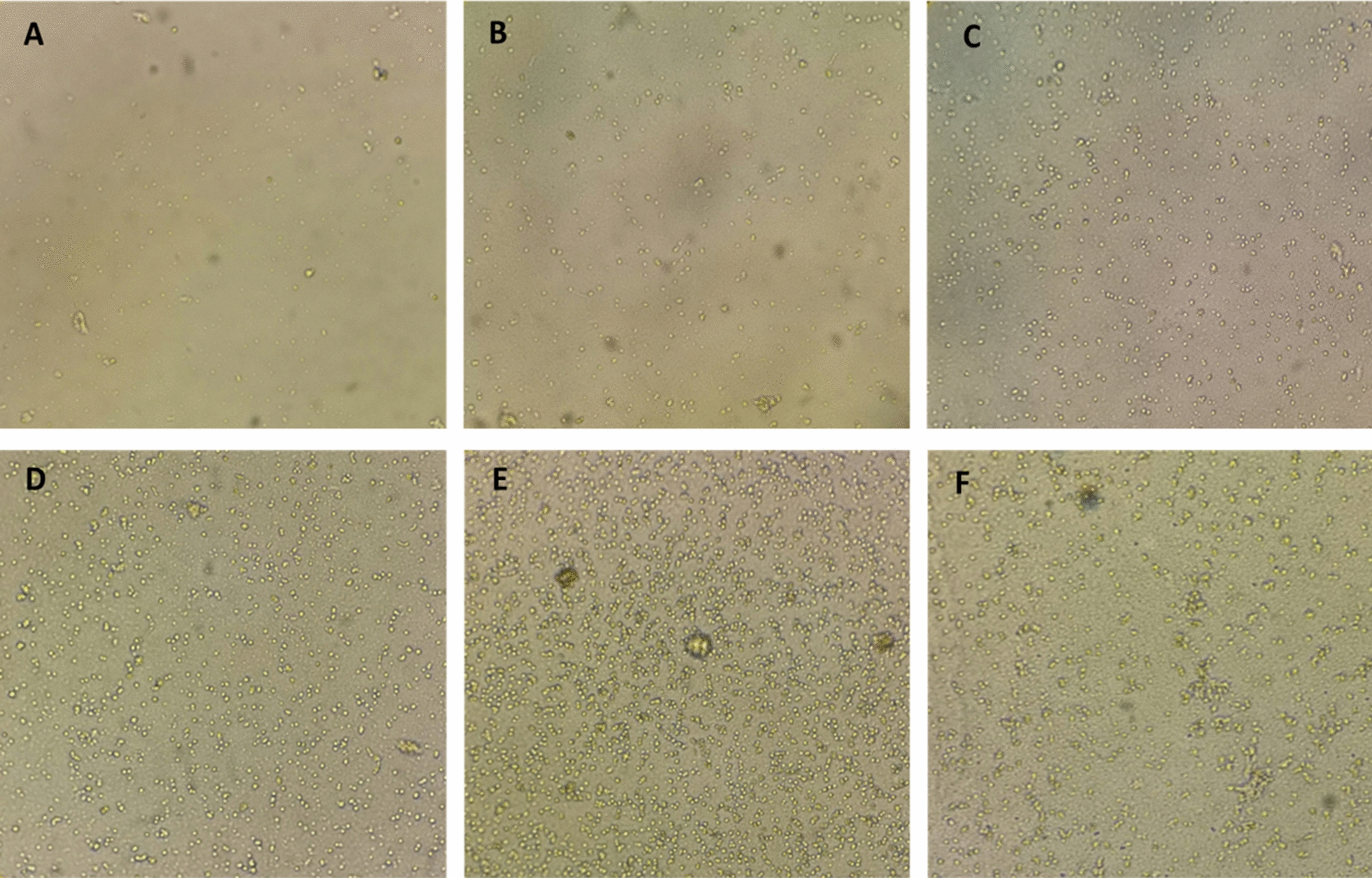
Inverted microscopy of *Echinococcus granulosus* germinative cell cultures at 7 (**a**), 14 (**b**), 20 (**c**), 30 (**d**) and 45 (**e**) days of cultivation. Note the aggregation behavior of the cells and initial monolayer formation at 60 days of culture (**f**)

Eighteen hours after the start of cultivation, the cells attached to the bottom of the plate and began to divide via binary division. The cells were round in shape and variable in size with an average diameter of approximately 2–4 µm. A gradual increase in the dividing cells was observed at 7 and 15 days after the start of cultivation (Fig. 1a, b), and a significant increase in the cell number was evident at 20, 30 and 45 days after incubation (Fig. 1c–e) so that at 45 days of cultivation the number of cells increased to 5 × 10^4^ cells/ml culture medium. However, at 60 days of incubation the number of cells had decreased and aggregation behavior was noticeable (Fig. 1f).

**Fig. 2:**
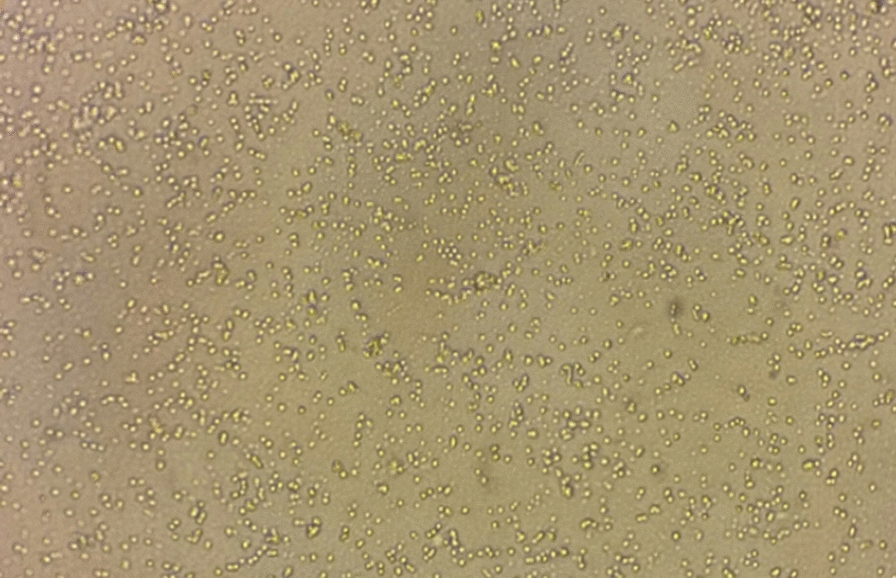
Inverted microscopy of *Echinococcus granulosus* germinative cell cultures, 10 months after plating

After passaging, the cells continue their growth, and gradual division and proliferation of the cells were evident over the time period of 1 to 60 days of incubation. Even though the cell growth was slow till 30 days, it was much higher between 30 and 60 days of incubation. However, the cultured cells in two plates remained viable for nearly 10 months, without any passaging (Fig. 2).

The chemical compositions of ZMEO identified by GC-MS analysis are shown in Table 1. A total of 52 compounds were identified, among which carvacrol (56.95%), *p*-cymene (7.49%), g-terpinene (7.12%), thymol (4.60%), a-pinene (3.58%) and carvacrol methyl ether (3.03%) were the major components.

**Table 1:** Chemical composition of *Zataria multiflora* essential oil identified by gas chromatography-mass spectroscopy

No.	Component	%	RI	No.	Component	%	RI
1	Tricyclene	0.012	922.1	27	a-Terpineol	0.458	1190
2	a-Thujene	0.585	925.4	28	*trans*-Dihydro carvone	0.078	1203
3	a-Pinene	3.583	930.9	29	Thymol methyl ether	0.063	1233
4	Camphene	0.189	951.1	30	Carvacrol methyl ether	3.029	1243
5	Thuja-2,4(10)-diene	0.009	956.6	31	Unknown	0.058	1254
6	Sabinene	0.011	972.4	32	*p*-Anisaldehyde dimethyl acetal	0.087	1256
7	b-Pinene	0.745	976.1	33	(E)-Anethole	0.179	1284
8	3-Octanone	0.249	984.9	34	Thymol	4.601	1290
9	Myrcene	1.524	990.4	35	Carvacrol	56.951	1299
10	3-Octanol	0.277	994.4	36	Thymol acetate	0.109	1354
11	a-Phellandrene	0.258	1001	37	Eugenol	0.056	1357
12	p-Mentha-1(7),8-iene	0.073	1004	38	Carvacrol acetate	1.984	1373
13	a-Terpinene	1.817	1016	39	(E)-Caryophyllene	1.958	1418
14	*p*-Cymene	7.497	1025	40	Unknown	0.069	1426
15	Limonene	0.627	1028	41	b-Copaene	0.066	1432
16	1,8-Cineole	0.223	1030	42	Aromadendrene	0.624	1437
17	(Z)-b-Ocimene	0.02	1036	43	Unknown	0.095	1441
18	(E)-b-Ocimene	0.061	1046	44	a-Humulene	0.15	1451
19	g-Terpinene	7.127	1059	45	allo-Aromadendrene	0.134	1458
20	*cis*-Sabinene hydrate	0.05	1066	46	b-Selinene	0.069	1486
21	Terpinolene	0.311	1088	47	d-Selinene	0.059	1489
22	Linalool	1.104	1098	48	Viridiflorene	0.718	1493
23	1-Octen-3-yl acetate	0.073	1111	49	b-Bisabolene	0.043	1507
24	3-Octanol acetate	0.033	1123	50	d-Cadinene	0.03	1522
25	Borneol	0.16	1164	51	Spathulenol	0.356	1575
26	Terpinen-4-ol	1.035	1176	52	Caryophyllene oxide	0.317	1581

*RI* retention index

The results of the *in vitro* lethal effect of ZMEO on the cultured germinative cells of *E. granulosus* are summarized in Table 2. As shown in this table, ZMEO with concentrations of 0.5 and 1 mg/ml (after 48 h), 2 mg/ml (after 12 h), 3 mg/ml (after 3 h) and 5 and 6 mg/ml (after 30 min) showed no lethal effect on the cultured germinative cells of *E. granulosus*. However, when used for a longer time or with higher concentrations, the lethality of the oil increased gradually. When the cells were treated with 3 mg/ml ZMEO, its lethal effect on the cells was 29 and 100% after 6 and 12 h, respectively. The lethal effect of 4 mg/ml ZMEO on the cells was 51% and 100% after 2 and 3 h, respectively. ZMEO at concentrations of 5 and 6 mg/ml killed all the cells after 1 h. However, 7 mg/ml ZMEO killed 45% and 100 of cells after 15 and 25 min, respectively. When the oil was used at 8 mg/ml concentration, its lethal effect was 46% and 100% only after 5 and 7 min, respectively. The obtained results evidently showed that the lethal effect of ZMEO on the germinative cells of *E. granulosus* was concentration and time dependent. Statistical analysis revealed a significant lethality of ZMEO on the cultured cells at concentrations of 2 mg/ml (after 24 h), 3 mg/ml (after 6 h), 4 mg/ml (after 2 h), 5 and 6 mg/ml (after 60 min), 7 mg/ml (after 25 min) and 8 mg/ml (after 5 min) (*P* < 0.05). The lethal effect of ZMEO was significantly higher than that of the positive control (albendazole) (*P* < 0.05) when the oil was applied at concentrations of 5 and 6 mg/ml (after 60 min), 7 mg/ml (after 25 min) and 8 mg/ml (after 5 min) (Table 2).

**Table 2:** Lethal effect of *Z. multiflora* essential oil (ZMEO) with different concentrations and at different exposure times on the cultured germinative cells of *Echinococcus granulosus*

Cons (mg/ml)	Lethal effect on the cultured cells (mean ± SD) (%)*				
	Time	ZMEO	Tween 80**	Albendazole (20 mg/ml)	Negative control
0.5	12 h	0.00^a^	0.00^a^	–	0.00^a^
	24 h	0.00^a^	0.00^a^	–	0.00^a^
	48 h	0.00^a^	0.00^a^	–	0.00^a^
1	12 h	0.00^a^	0.00^a^	–	0.00^a^
	24 h	0.00^a^	0.00^a^	–	0.00^a^
	48 h	0.00^a^	0.00^a^	–	0.00^a^
2	12 h	0.00^a^	0.00^a^	–	0.00^a^
	24 h	20.05 ± 1.86^a^	0.00^b^	–	0.00^b^
	48 h	40.32 ± 2.11^a^	0.00^b^	–	0.00^b^
3	3 h	0.00^a^	0.00^a^	–	0.00^a^
	6 h	29.10 ± 3.63^a^	0.00^b^	–	0.00^b^
	12 h	100 ± 0.00^a^	0.00^b^	–	0.00^b^
4	2 h	51.75 ± 4.47^a^	0.00^b^	0.00^b^	0.00^b^
	3 h	100 ± 0.00^a^	0.00^b^	–	0.00^b^
5	30 min	0.00^a^	0.00^a^	0.00^a^	0.00^a^
	60 min	100 ± 0.00^a^	0.00^b^	0.00^b^	0.00^b^
6	30 min	0.00^a^	0.00^a^	0.00^a^	0.00^a^
	60 min	100 ± 0.00^a^	0.00^b^	0.00^b^	0.00^b^
7	15 min	45.90 ± 3.00^a^	0.00^b^	0.00^b^	0.00^b^
	25 min	100 ± 0.00^a^	0.00^b^	0.00^b^	0.00^b^
8	5 min	46.33 ± 3.79^a^	0.00^b^	0.00^b^	0.00^b^
	7 min	100 ± 0.00^a^	0.00^b^	0.00^b^	0.00^b^

ZMEO: *Zataria multiflora* essential oil, Cons: concentrations, h: hour, min: minutes

*Different letters show significant difference in each row

**Tween 80 was used at twice the concentration of ZMEO

## Discussion

*Echinococcus granulosus* (*s.l.*) is the causative agent for CE, which is an important disease; hence, study on various features of this parasite such as its biology, pathogenesis, diagnosis and treatment is important [14]. There are two alternative tools for the study on the larval form of *E. granulosus*: the culture of parasite in the laboratory and establishment of the infection in laboratory animals [15]. However, *in vivo* experiments are costly, time consuming and ethically problematic [21].

Many attempts have been made to establish cell cultures from nematodes [22, 23], trematodes [24] and cestodes [17, 25–29]. The wall of the larval form of *E. granulosus* (CE cyst) has two layers: an acellular external laminated layer (made of polysaccharides) and an internal germinal layer consisting of multiplying cells, which may differentiate into protoscoleces or daughter cysts. Proliferating cells of the germinal layer may be used for *in vitro* culture [17].

Compared to the vast knowledge available on the morphological features of CE cysts, little information is available on the molecular structure of the germinal layer of the parasite. The germinal layer of CE cysts with high ATPase activity plays a significant role in the growth and survival of the cyst [30]. The germinal layer is joined with the brood capsules’ wall as well as with the tegument of the protoscoleces [31]. On the other hand, the germinal layer structure and organization are similar to the tegument of the protoscoleces, the brood capsules’ wall and also the tegument of the adult form of the parasite [32].

According to the critical role of the germinal layer in the growth and survival of the cyst, it is wise to evaluate the effect of anti-echinococcal agents on the germinative cells of this layer. Hence, availability of *E. granulosus* germinative cells is quite relevant for development of more effective anti-CE drugs [18].

In the present study, we attempted to evaluate the lethal effect of ZMEO directly on the germinative cells of *E. granulosus*. The primary need for this study was the accessibility of the live cultured germinative cells of the parasite. That is why we first tried to establish the required cell cultures for our experiments.

Several attempts have been to establish primary cell cultures from the germinative cells of *E. granulosus* [19]. The complexity of the *E. granulosus* biological cycle, difficulty in production of appropriate *in vitro* culture conditions and inadequate cell isolation methods may be considered the main factors related to failure in establishing primary cell cultures from *E. granulosus* germinative cells [29]. However, our efforts in cultivation of *E. granulosus* germinative cells were successful. Even though the cells’ growth was slow for several days after the start of cultivation, almost 3 weeks later, the number of cells was considerable, and 45 days after the start of cultivation, the cell concentration was suitable for the desired experiments (5 × 10^4^ cells/ml).

We observed many cells while they had the dividing status. Proliferating *E. granulosus* germinative cells with high protein synthesis potential and high proliferating activity differentiate into protoscoleces of the hydatid cyst [33]. These cells are also capable of producing new cysts [19]. In the current study, the inner surface of germinal layers was scraped gently by a scraper, and the obtained pellets were trypsinized for cell isolation. As previously reported by Albani et al. [19], we also obtained cultures with no contamination with host cells.

Albani et al. [19] succeeded in maintaining the isolated cells of the germinal layer of CE cysts *in vitro* for at least 4 months. They stated that “The cells grew for over 10 passages without morphological or biochemical changes.” In the present study, the cells remained alive for at least 60 days, which was enough for *in vitro* evaluation of the lethal effect of ZMEO. In addition, the results of cell passaging were also satisfactory. The cells were passaged four times with 60-day intervals, and they were still alive and dividing after the fourth passage. However, in the current study, the cells of two culture plates remained viable for nearly 10 months without any passaging. This may be attributed to the high proliferative potential of *E. granulosus* germinative cells.

The present study was done to evaluate the lethal effect of ZMEO on the germinative cells of *E. granulosus*. Luckily, the results were hopeful. Antidiabetic, anti-aphthous, anti-nociceptive, anti-inflammatory, antiprotozoal, antimicrobial and antifungal activities of ZMEO have been previously documented [34]. *In vitro* high scolicidal activity has been previously reported for the methanolic extract [35] and aromatic water of *Z. multiflora* [36]. In addition, anti-hydatid properties of the methanolic extract [37], aromatic water [38] and essential oil [39–42] of *Z. multiflora* have been reported in earlier *in vivo* studies.

In the present study, the lethal effect of ZMEO on the germinative cells of *E. granulosus* at concentrations of 3, 4, 5 and 6 mg/ml was 100% after 12, 3, 1 and 1 h of exposure, respectively. The lethal efficacy of the oil was much higher at concentrations of 7 and 8 mg/ml, which means that ZMEO killed all the cells with these concentrations after only 25 and 7 min, respectively. To the best of our knowledge, this is the first report that describes the lethal effect of ZMEO on the germinative cells of *E. granulosus*.

Benzimidazoles, as the most familiar therapeutic drugs for CE, only have a static effect on the parasite; therefore, complete cure may not be achieved after administration of albendazole, even with high doses and for long periods of time [43]. On the other hand, long-term albendazole therapy with high doses may be accompanied by adverse effects in treated patients [2, 4, 44, 45]. The dynamic nature and complexity of CE make its treatment problematic [46]. Many efforts are being made to introduce novel therapeutic agents for treatment of the disease worldwide.

In accordance with our study, in previous chemical analysis by GC and GC–MC, thymol and carvacrol have been found to be the main components of ZMEO [34, 40, 47, 48]; interestingly, both have scolicidal power and anti-CE properties [49–51].

Both thymol and carvacrol, as the lipophilic compounds, could simply enter the cell membranes, altering their permeability and causing the release of cellular contents [34]. This property may elucidate the lethal effect of ZMEO on germinative *E. granulosus* cells. In earlier studies, the antioxidant, hepatoprotective and immunostimulatory activities of ZMEO have been documented [34, 52]. Previous studies also confirmed that thymol and carvacrol as the main components of ZMEO are safe compounds [53].

Regarding the above issues, the lethal effect of ZMEO on the germinative cells of *E. granulosus* might be considered an encouraging step forward in the medical treatment of CE using an herbal product.

## Conclusions

Forty-five days after the start of cultivation, the germinative cell concentration was suitable for evaluation of the *in vitro* lethal effect of ZMEO on the cultured cells. ZMEO at concentrations of 7 and 8 mg/ml killed all the cells after only 25 and 7 min, respectively. The results of this study revealed that the oil effect on the cells was concentration and time dependent. These findings provide an opportunity for introduction of a novel, more effective and safer therapeutic agent for treatment of CE. However, further studies are required in laboratory animals before recommendation of this treatment modality in humans.

## Data Availability

All relevant data are within the main paper.

## Abbreviations

CE: Cystic echinococcosis
ZMEO: *Zataria multiflora* essential oil
GC-MS: Gas chromatography-mass spectrometry
PAIR: Puncture, aspiration, injection, re-aspiration
FBS: Fetal bovine serum
PBS: Phosphate-buffered saline
EDTA: Ethylenediaminetetraacetic acid
